# Survival analysis and prognosis of patients with breast cancer with pleural metastasis

**DOI:** 10.3389/fonc.2023.1104246

**Published:** 2023-05-01

**Authors:** Sumei Li, Chao Li, Wenna Shao, Xiaoyu Liu, Luhao Sun, Zhiyong Yu

**Affiliations:** ^1^ College of Traditional Chinese Medicine, Shandong University of Traditional Chinese Medicine, Jinan, China; ^2^ Department of Breast Surgery, Shandong Cancer Hospital and Institute, Shandong First Medical University and Shandong Academy of Medical Sciences, Jinan, China; ^3^ First Clinical Medical College, Shandong University of Traditional Chinese Medicine, Jinan, China

**Keywords:** breast cancer, metastatic breast cancer, first metastatic site, pleural metastasis, survival, prognostic factors, nomogram model

## Abstract

**Background:**

Breast cancer (BC) is the most common malignant cancer. The prognosis of patients differs according to the location of distant metastasis, with pleura being a common metastatic site in BC. Nonetheless, clinical data of patients with pleural metastasis (PM) as the only distant metastatic site at initial diagnosis of metastatic BC (MBC) are limited.

**Patient cohort and methods:**

The medical records of patients who were hospitalized in Shandong Cancer Hospital between January 1, 2012 and December 31, 2021 were reviewed, and patients eligible for the study were selected. Survival analysis was conducted using Kaplan–Meier (KM) method. Univariate and multivariate Cox proportional-hazards models were used to identify prognostic factors. Finally, based on these selected factors, a nomogram was constructed and validated.

**Results:**

In total, 182 patients were included; 58 (group A), 81 (group B), and 43 (group C) patients presented with only PM, only lung metastasis (LM), and PM combined with LM, respectively. The KM curves revealed no significant difference in overall survival (OS) among the three groups. However, in terms of survival after distant metastasis (M-OS), the difference was significant: patients with only PM exhibited the best prognosis, whereas those with PM combined with LM exhibited the worst prognosis (median M-OS: 65.9, 40.5, and 32.4 months, respectively; P = 0.0067). For patients with LM in groups A and C, those with malignant pleural effusion (MPE) exhibited significantly worse M-OS than those without MPE. Univariate and multivariate analyses indicated that primary cancer site, T stage, N stage, location of PM, and MPE were independent prognostic factors for patients with PM without other distant metastasis. A nomogram prediction model incorporating these variables was created. According to the C-index (0.776), the AUC values of the 3-, 5-, and 8-year M-OS (0.86, 0.86, and 0.90, respectively), and calibration curves, the predicted and actual M-OS were in good agreement.

**Conclusion:**

BC patients with PM only at the first diagnosis of MBC exhibited a better prognosis than those with LM only or PM combined with LM. We identified five independent prognostic factors associated with M-OS in this subset of patients, and a nomogram model with good predictive efficacy was established.

## 1 Introduction

Breast cancer (BC) is the cancer with the highest prevalence worldwide (1) and the leading cause of cancer-related deaths among females (2). BC has a tendency for distant metastasis (3), and the majority of BC-related deaths are due to metastasis (4). BC exhibits heterogeneity in metastasis and prognosis (1). Even though patients with distant metastases are all defined as MBC (5), different sites of metastasis have variable impacts on clinical outcomes (6), and the prognosis varies greatly. The metastatic sites should be taken into consideration when assessing prognosis and making therapeutic strategies for patients with MBC (5).

The lung and pleura are among the most common metastatic sites of BC (7). Cummings MC et al. performed an autopsy examination of women who died of BC and found that the most common organs involved were lung/pleura, followed by bone, liver and non-axillary lymph nodes (8). The lung is generally accepted as one of the primary target visceral organs of BC metastasis (1), and is anatomically related to pleura. Lung metastasis (LM) is the most common accompanied organ metastasis site of pleural metastasis (PM) in BC. Some studies on MBC did not distinguish between LM and PM (8–11). Thus when interpreting these data, it must be noted that LM are referred to as either including or excluding PM (12). The site of first distant metastasis correlates with the survival of patients with BC (13, 14). And lung metastasis (LM) is of particular attention because of its high morbidity and association with high mortality of patients (15). PM usually manifested as pleural nodulations or pleural thickening (16), with or without malignant pleural effusion (MPE) (17). However, PM often goes unnoticed until the appearance of MPE. There is little evidence regarding the prognosis of patients with MBC when pleura is the first recurrence site. In the present study, we wanted to explore the prognostic differences between pleural and lung when serving as the first site of distant metastasis after radical surgery for primary BC, which could help to supplement the vacancy of current data.

MPE is a common manifestation of PM (18) and a frequent complication during the course of MBC (19). Approximately 11% of the patients with BC eventually present with symptomatic pleural effusions; at autopsy, 36%–65% of patients retrospectively suffered from this condition (20, 21). Although MPE is rarely the initial manifestation of cancer (20), it carries a significant symptom burden (22) and is considered to be associated with a dismal prognosis (23). However, not all PM was accompanied by MPE (24), especially when the initial diagnosis of MBC. Therefore, we hypothesized that among BC patients with PM, the presence or absence of MPE would lead to different prognoses. The diversity in prognosis of BC patients is caused by the combined effect of multiple pathological factors. An understanding of prognostic factors is imperative for individualization of prognosis in patients with BC. However, the prognostic factors in BC patients with PM are unclear, particularly when no other distant metastases exist. In addition, the prognosis plays a central role for patients with BC and oncologists to choose optimally treatment in this era of individualized therapy (25). Therefore, an accurate prediction model is needed for this subset of patients.

Over the past few years, nomograms have been widely recognized as a predictive method for several diseases, including BC (26). Nomograms can generate an individual probability of a clinical event by integrating diverse determinant variables and meet the requirement for biologically and clinically integrated models (27). Evidence-based guidelines suggest using conservative treatments in patients with limited life expectancy, whereas they suggest offering more aggressive treatment modalities for patients with better prognosis. Real-world data can inform the outcome comparisons (28). Our study aimed to investigate whether patients with BC in whom PM was the primary event of recurrence exhibit a prognosis different than those with LM. Furthermore, we explored the prognostic factors and created a nomogram model, which can aid physicians in better evaluating the patient’s prognosis and selecting patients for different treatment tactics.

## 2 Patients and methods

### 2.1 Study population and variables

**Figure 1:**
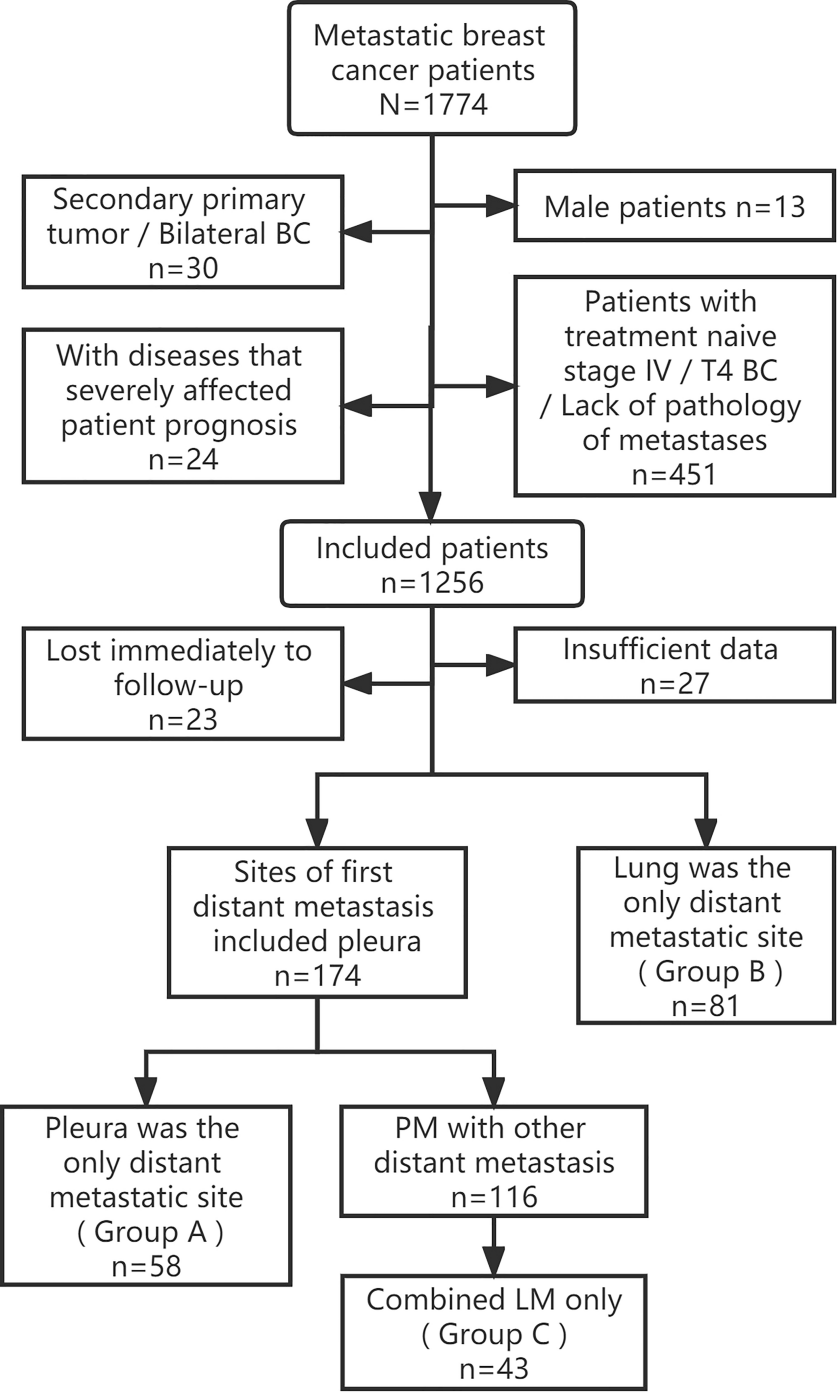
Flow chart of patient selection. BC, Breast cancer; PM, Pleural metastasis; LM, Lung metastasis.

This was a single-center retrospective cohort study. Patients with MBC confirmed by pathology who were consecutively hospitalized at Shandong Cancer Hospital between January 1, 2012 and December 31, 2021, were included in this study. The inclusion criteria according to primary BC were as follows: (1) female patients who had undergone radical surgery for BC; (2) T stage was 1–3; and (3) unilateral BC. Patients with other malignancies or diseases that severely affected the patient’s survival and prognosis were eliminated. These diseases include acute myocardial infarction/congestive heart failure, acute cerebrovascular disease, chronic obstructive pulmonary disease, irreversible severe renal/hepatic impairment (such as severe hepatitis, cirrhosis…), serious mental illness, diabetes mellitus with severe complications. Tracing patient’s clinical data, and the metastatic sites at the first diagnosis of MBC after surgery were determined. Only those with PM or LM at first MBC diagnosis were further screened, 182 patients were finally included in this study and pertinent data were updated retrospectively using current tumor classification criteria. The Flow chart of patient selection was shown in **Figure 1**.

The diagnosis of PM was based on pleural biopsy results, imaging, pleural fluid analysis, and medical thoracoscopy. Cancer staging of the primary cancer was based on the TNM staging system by the 8th American Joint Committee on Cancer (AJCC). HER2 was determined locally by IHC/FISH and determined positive by 3+ staining or FISH positivity (29). Cancers with estrogen receptor-positive (ER+) and/or progesterone receptor-positive (PR+) were considered hormone receptor-positive (HR+), while ER-negative (ER-) and PR-negative (PR-) were considered HR-negative (HR-). Distant metastasis-free interval was defined as the period after radical surgery till the first diagnosis of MBC. Medical attention due to symptoms refers to the diagnosis of MBC was because of symptoms such as chest pain, dyspnea and thoracic pressure, rather than regular follow-up examinations. OS and survival time after distant metastasis (M-OS) were defined as the time from the diagnosis of BC or distant metastasis to death, respectively. The follow-up cut-off was July 31, 2022. If the patient was alive at the last censored follow-up, we considered her to have not reached the study endpoint. Our study was approved by the Shandong Cancer Hospital Ethical Committee.

### 2.2 Statistical analysis and model construction

Chi-square tests were used to compare the clinicopathological characteristics among groups. Comparisons of continuous variables were performed using ANOVA. The Kaplan–Meier (KM) method was used to calculate the survival end-points (OS and M-OS), and the log-rank test was conducted to assess the differences among subgroups. The factors independently associated with M-OS of patients with PM were assessed using univariate and multivariate Cox regression analyses, and hazard ratios (HRs) with 95% confidence intervals (CIs) were calculated. A two-sided P value < 0.05 was considered significant. A prediction nomogram based on the results of multivariate logistic regression analysis was developed using the “rms” package. The concordance index (C-index) was generated to measure the predictive accuracy and discrimination capabilities. Receiver operating characteristic (ROC) curves were depicted and the predictive accuracy was examined with the area under the curve (AUC). A calibration curve was plotted to test the association between the expected probabilities and observed outcome frequencies.

## 3 Results

### 3.1 Patient baseline

We obtained the clinical data of these 182 patients and followed them up. PM was the primary event at first MBC diagnosis in 58 (31.9%) patients (group A), 81 (44.5%) patients (group B) had LM without other distant metastases, and 43 (23.6%) patients had LM and PM (group C). The baseline features of these individuals according to metastatic sites are given in **Table 1**. The median age of patients at initial BC diagnosis was 42 years (range, 23–71 years) and most patients were premenopausal (73.1%). In terms of therapy, the majority of patients (91.2%) did not receive neoadjuvant chemotherapy and 151 (83.0%) patients underwent a mastectomy. Most patients were at histopathological grade II (58.2%) or T2 stage (56.6%). Moreover, 23.1% of patients were at the N0 stage, 36.8% at N1, 20.3% at N2, and 19.8% at N3. Luminal B was the most common molecular subtype (33.5%), followed by luminal A (26.9%), triple-negative (23.6%), and HER-2-enriched type (16.0%). After surgery, 180 (98.9%) patients received chemotherapy and 79 (43.4%) received radiotherapy. There was no significant difference in the distribution of the described variables among the three groups (**Table 1**).

**Table 1:** Baseline characteristics of the entire cohort.

Characteristic	Total (N=182)	Group A (N=58)	Group B (N=81)	Group C (N=43)	P Value
**Age at BC diagnosis (years)**					0.154
Median (range)	42 (23-71)	43 (26-71)	41 (28-65)	44 (23-70)	
Mean (SD)	44 (9.4)	46 (10.3)	43 (8.2)	42 (10.0)	
**Menstrual status**					0.820
premenopause	133 (73.1)	42 (72.4)	58 (71.6)	33 (76.7)	
menopause	49 (26.9)	16 (27.6)	23 (28.4)	10 (23.3)	
**Neoadjuvant Chemotherapy**					0.862
Received	16 (8.8)	5 (8.6)	8 (9.9)	3 (7.0)	
Not received	166 (91.2)	53 (91.4)	73 (90.1)	40 (93.0)	
**Surgery type**					0.812
Mastectomy	151 (83.0)	48 (82.8)	66 (81.5)	37 (86.0)	
Lumpectomy	31 (17.0)	10 (17.2)	15 (18.5)	6 (14.0)	
**Molecular Subtype**					0.539
Luminal A	49 (26.9)	16 (27.6)	21 (25.9)	12 (27.9)	
Luminal B	61 (33.5)	23 (39.7)	23 (28.4)	15 (34.9)	
Triple negative	43 (23.6)	9 (15.5)	25 (30.9)	9 (20.9)	
HER2 enriched	29 (16.0)	10 (17.2)	12 (14.8)	7 (16.3)	
**Histopathological grading**					0.863
I	24 (13.2)	7 (12.1)	11 (13.6)	6 (14.0)	
II	106 (58.2)	35 (60.3)	44 (54.3)	27 (62.8)	
III	52 (28.6)	16 (27.6)	26 (32.1)	10 (23.3)	
**T category**					0.549
T1	61 (33.5)	15 (25.9)	29 (35.8)	17 (39.5)	
T2	103 (56.6)	38 (65.5)	43 (53.1)	22 (51.2)	
T3	18 (9.9)	5 (8.6)	9 (11.1)	4 (9.3)	
**N category**					0.821
N0	42 (23.1)	14 (24.1)	15 (18.5)	13 (30.2)	
N1	67 (36.8)	19 (32.8)	33 (40.7)	15 (34.9)	
N2	37 (20.3)	13 (22.4)	16 (19.8)	8 (18.6)	
N3	36 (19.8)	12 (20.7)	17 (21.0)	7 (16.3)	
**Adjuvant Chemotherapy**					0.238
Done	180 (98.9)	58 (100)	79 (97.5)	43 (100)	
Not done	2 (1.1)	0 (0)	2 (2.5)	0 (0)	
**Radiotherapy**					0.956
Done	79 (43.4)	26 (44.8)	35 (43.2)	18 (41.9)	
Not done	103 (56.6)	32 (55.2)	46 (56.8)	25 (58.1)	

Group A, PM without other distant metastases; Group B, LM without other distant metastases; Group C, LM and PM without other distant metastases. Data are presented as No. (%) or median (range), unless otherwise indicated. SD, standard deviation.

### 3.2 Survival analysis

**Figure 2:**
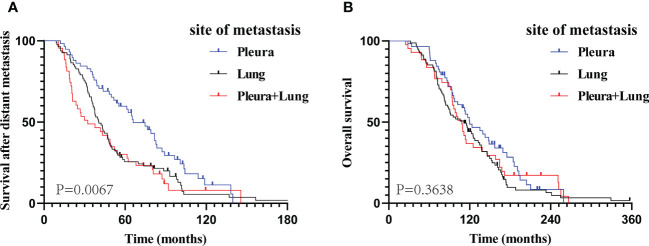
The Kaplan–Meier curve analysis of study cohorts. M-OS **(A)** and OS **(B)** curves according to different metastatic sites at the time of diagnosed of metastatic breast cancer. M-OS, survival after distant metastasis; OS, overall survival.

At the end of the follow-up period, 158 (86.8%) patients died. Meanwhile, 13 (22.4%), 7 (8.6%), and 4 (9.3%) patients were alive in groups A, B, and C, respectively. The 3-, 5-, and 8-year cumulative M-OS rates of patients in groups A, B, and C, respectively, were 79.3%, 61.7%, and 48.8%; 53.4%, 23.5%, and 30.2%; and 20.7%, 12.3%, and 4.6%. The prognosis of patients with only PM (group A) was significantly better than that of patients with only LM (group B) or LM with PM (group C) in terms of M-OS (median M-OS: 65.9 vs. 40.5 vs. 32.4 months, P = 0.0067; **Figure 2A**); however, the difference in their OS was not significant (median OS: 119.8 vs. 111.2 vs. 108.2 months, P = 0.3638; **Figure 2B**). The M-OS was significantly prolonged in group A compared with that in groups B (median M-OS: 65.9 vs 40.5 months, P = 0.0060; **Supplemental Figure 1A**) or C (median M-OS: 65.9 vs 32.4 months, P = 0.0077; **Supplemental Figure 1B**). There was no significant difference in M-OS between groups B and C (median M-OS: 40.5 vs 32.4 months, P = 0.3789; **Supplemental Figure 1C**). Additionally, there was no significant difference in OS between groups A and B, groups A and C, and groups B and C (median OS: 119.8 vs. 111.2 months, P = 0.1223; 119.8 vs. 108.2 months, P = 0.5760; 111.2 vs. 108.2 months, P = 0.6102, respectively; **Supplemental Figures 1D–F**).

**Figure 3:**
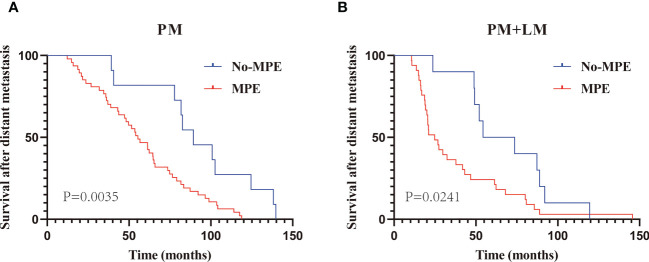
The Kaplan–Meier curve analysis of M-OS in PM with or without MPE. Patients with only PM **(A)**; patients with PM and LM **(B)**. PM, Pleural metastasis; MPE, Malignant pleural effusion; LM, Lung metastasis.

Given the high incidence of MPE in patients with PM (81.0% in group A and 76.7% in group C), we compared the M-OS between patients with and without MPE within the two groups. We observed a significant difference in M-OS (median M-OS: 55.4 vs. 89.3 months in group A, P = 0.0035; 24.8 vs. 64.0 months in group C, P = 0.0241) between patients with and without MPE (**Figures 3A, B** **)**.

### 3.3 Characteristics of PM patients without other distant metastasis

Then, we analyzed the clinicopathological features of PM patients without other distant metastasis. Overall, 35 of the 58 patients (60.3%) were < 45 years at initial BC diagnosis. A total of 48.3% and 51.7% of the cancers were lateralized to the left and right, respectively, and most were located in the inner quadrant of the breast (46.6%). As for local treatment, mastectomy was performed in 82.8% of patients. At initial BC diagnosis, these patients with a high proportion of AJCC stage III, T2 stage, and pathohistological grade II, corresponding to 48.3%, 65.5%, and 60.3%, respectively. The majority of cancers were HR-positive and HER2-negative (HR+HER2-) (67.2%), with the highest frequency in the luminal B subtype (39.7%). Overall, 55.2%, 77.6%, and 15.5% of patients received radiotherapy, endocrine therapy, and anti-HER2 therapy, respectively. In total, 40 (69.0%) patients were diagnosed with distant metastasis within 5 years of radical surgery. Overall, medical attention due to symptoms was recorded in 17 (29.3%) patients, and 14 (24.1%) patients had a chest wall recurrence. Most PM (79.3%) were located ipsilateral to the primary BC, and 47 (81.0%) patients presented with MPE. Supraclavicular lymph node metastasis was observed in 20 (34.5%) patients (6 patients were identified at the time of surgery for the primary BC, and 14 were diagnosed concomitantly with the PM). Detailed patient characteristics are given in **Table 2**.

**Table 2:** Characteristics of patients with PM as the only site of distant metastasis at first MBC diagnosis.

Variable	Number	Percent
Age at initial BC diagnosis (years)		
<45	35	60.3%
≥45	23	39.7%
Laterality		
Left	28	48.3%
Right	30	51.7%
Primary tumor site		
Outer quadrant	18	31.0%
Inner quadrant	27	46.6%
The areolar region/central axis	13	22.4%
Surgery type		
Mastectomy	48	82.8%
Lumpectomy	10	17.2%
Two diameter ratio		
<1.4	28	48.3%
≥1.4	30	51.7%
AJCC stage at initial BC diagnosis		
I	7	12.1%
II	23	39.6%
III	28	48.3%
T category of primary BC		
T1	15	25.9%
T2	38	65.5%
T3	5	8.6%
N category of primary BC		
N0	14	24.1%
N1	19	32.8%
N2	13	22.4%
N3	12	20.7%
Histopathological Grade of primary BC		
I	7	12.1%
II	35	60.3%
III	16	27.6%
Molecular Subtype of metastases		
Luminal A	16	27.6%
Luminal B	23	39.7%
Triple negative	9	15.5%
HER2 enriched (HR +/HR -)	10	17.2%
Subtype		
HR+Her2+	6	10.3%
HR+Her2-	39	67.2%
HR-Her2+	4	6.9%
HR-Her2-	9	15.5%
ER		
Negative	13	22.4%
Positive	45	77.6%
PR		
Negative	19	32.8%
Positive	39	67.2%
Ki-67		
≤20%	36	62.1%
>20%	22	37.9%
Radiotherapy		
Received	32	55.2%
Not received	26	44.8%
Endocrine therapy		
Received	45	77.6%
Not received	13	22.4%
Anti-HER2 therapy		
Received	9	15.5%
Not received	49	84.5%
Distant metastasis free interval		
≤ 5 yrs.	40	69.0%
> 5 yrs.	18	31.0%
Chest wall recurrence		
Yes	14	24.1%
No	44	75.9%
Age at diagnosis of MBC (years)		
<50	29	50.0%
≥50	29	50.0%
Female hormone levels		
Premenopausal status	27	46.6%
Menopausal status	31	53.4%
hether the patient came to medical attention because of symptoms		
Yes	17	29.3%
No	41	70.7%
Location of PM		
Ipsilateral	46	79.3%
Contralateral/Bilateral	12	20.7%
MPE		
Yes	47	81.0%
No	11	19.0%
Supraclavicular lymph node metastasis		
Yes	20	34.5%
No	38	65.5%

BC, breast cancer; PM, pleural metastasis; MPE, malignant pleural effusion; MBC, metastatic breast cancer; HR+, hormone receptor-positive; HR-, hormone receptor-negative.

### 3.4 Screening of prognostic variables

**Figure 4:**
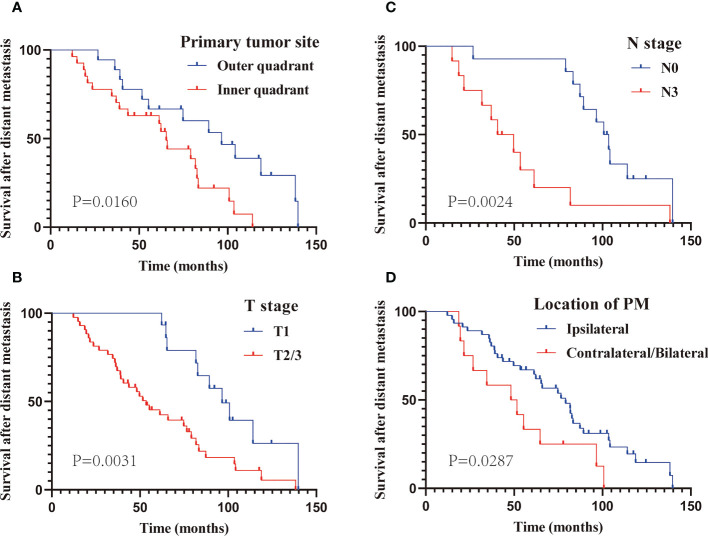
The Kaplan–Meier curve analysis of M-OS in subgroups based on multivariate analysis. Subgroup of primary site **(A)**; T stage **(B)**; N stage **(C)**; location of PM **(D)**. PM, Pleural metastasis.

The prognostic factors of patients with only PM at first MBC diagnosis assessed using Cox regression analyses are presented in **Table 3**. It is worth mentioning that the AJCC stage, to some extent, corresponds to the T and N stage categorization. Thus, to avoid repetition, only T and N stage classifications were included in our univariate analysis. Six variables (primary cancer site, T stage, N stage, molecular subtype, location of PM, and MPE) that were significantly associated with M-OS (P < 0.05) in univariate analysis were further included in the multi-factor Cox regression model. Based on the multivariate analysis, we ultimately ascertained that primary cancer in inner quadrant (vs. outer quadrant; HR: 3.65; 95% CI: 1.52–8.79; P = 0.004), T2/3 stage (vs. T1 stage; HR: 2.68; 95% CI: 1.11–6.43; P = 0.028), N3 stage (vs. N0 stage; HR: 5.30; 95% CI: 1.40–19.99; P = 0.014), PM located contralateral/bilateral to the primary BC (vs. ipsilateral; HR: 3.41; 95% CI: 1.42–8.19; P = 0.006), and MPE (vs. without MPE; HR: 4.42; 95% CI = 1.39–14.05; P = 0.012) were significantly correlated with poor M-OS of patients with PM (**Table 3**). Additionally, the KM curves confirmed the above statistical findings. Patients whose primary cancer was located in the inner quadrant were more likely to survive for a shorter time than those whose primary cancer was located in the outer quadrant (P = 0.0160; **Figure 4A**). Survival rates declined with high T stage (T2/3 vs. T1 stage, P = 0.0031; **Figure 4B**) and N stage (N3 vs. N0 stage, P = 0.0024; **Figure 4C**). Patients whose PM was located ipsilateral to the primary BC and without MPE tended to have a high survival probability (location of PM: P = 0.0287, **Figure 4D**; MPE: P = 0.0035, **Figure 3A**). In summary, primary cancer site, T stage, N stage, location of PM, and MPE were significant factors that were associated with M-OS.

**Table 3:** Cox analysis of prognostic factors in patients with PM as the only site of distant metastasis at first diagnosis of MBC.

Variable	Univariable analysis		Multivariable analysis	
	HR* (95% CI)	p value	HR *(95% CI)	p value
**Age at initial BC diagnosis (yrs.) (**≥45 vs. <45**)**	1.47 (0.79-2.74)	0.223	–	–
**Primary tumor site (**vs. Outer quadrant**)**				0.014
Inner quadrant	2.59 (1.18-5.65)	0.017	3.65 (1.52-8.79)	0.004
The areolar region/central axis	3.35 (1.35-8.29)	0.009	2.07 (0.70-6.13)	0.189
**Surgery (**Breast-conserving surgery vs. Mastectomy**)**	0.76 (0.35-1.66)	0.489	–	–
**Two diameter ratio (**≥1.4 vs. <1.4**)**	1.42 (0.78-2.61)	0.254	–	–
**T Stage of primary BC (**T2/3 vs. T1**)**	2.94 (1.40-6.20)	0.005	2.68 (1.11-6.43)	0.028
**N Stage of primary BC (**vs. N0**)**				0.078
N1	2.49 (1.06-5.88)	0.037	1.82 (0.58-5.76)	0.307
N2	4.35 (1.67-11.36)	0.003	3.14 (0.86-11.49)	0.084
N3	4.02 (1.65-9.82)	0.002	5.30 (1.40-19.99)	0.014
**Histopathological Grade of primary BC (**vs. I)				
II	1.60 (0.61-4.22)	0.342	–	–
III	2.24 (0.78-6.43)	0.134	–	–
**Molecular Subtype of metastases (**vs. Luminal A**)**				0.644
Luminal B	3.86 (1.74-8.58)	0.001	1.50 (0.52-4.32)	0.449
HER2 enriched (HR +/HR -)	1.68 (0.63-4.45)	0.300	1.05 (0.30-3.68)	0.938
Triple negative	2.25 (0.78-6.50)	0.134	0.75 (0.24-2.38)	0.625
**ER (**P vs. N**)**	1.51 (0.67-3.40)	0.323	–	–
**PR (**P vs. N**)**	0.99 (0.51-1.95)	0.990	–	–
**HER-2 (**P vs. N**)**	1.17 (0.48-2.81)	0.732	–	–
**Radiotherapy (**Not done vs. Done**)**	1.24 (0.68-2.28)	0.484	–	–
**Distant metastasis free interval (**> 5 yrs. vs. ≤ 5 yrs.**)**	0.56 (0.28-1.11)	0.098	–	–
**Chest wall recurrence (**Yes vs. ≤ No)	0.91 (0.45-1.84)	0.784	–	–
**Age at diagnosis of MBC (yrs.) (**≥50 vs. <50)	1.10 (0.60-2.02)	0.749	–	–
**Female hormone levels (**Menopausal status vs. Premenopausal status)	1.21 (0.66-2.21)	0.537	–	–
**Whether the patient came to medical attention because of symptoms (**Yes vs. No)	1.61 (0.82-3.14)	0.166	–	–
**Location of PM (**Contralateral/Bilateral vs. Ipsilateral)	2.15 (1.07-4.33)	0.033	3.41 (1.42-8.19)	0.006
**MPE (**Yes vs. No)	3.01 (1.24-7.28)	0.015	4.42 (1.39-14.05)	0.012
**Supraclavicular lymph node metastasis (**Yes vs. No)	1.55 (0.80-2.99)	0.192	–	–

-, negative; HR*, hazard ratio; BC, breast cancer; MBC, metastatic breast cancer; HR+, hormone receptor-positive; HR-, hormone receptor-negative; PM, pleural metastasis; MPE, malignant pleural effusion.

### 3.5 Construction and validation of a 3-, 5-, and 8-year M-OS predicting nomogram

**Figure 5:**
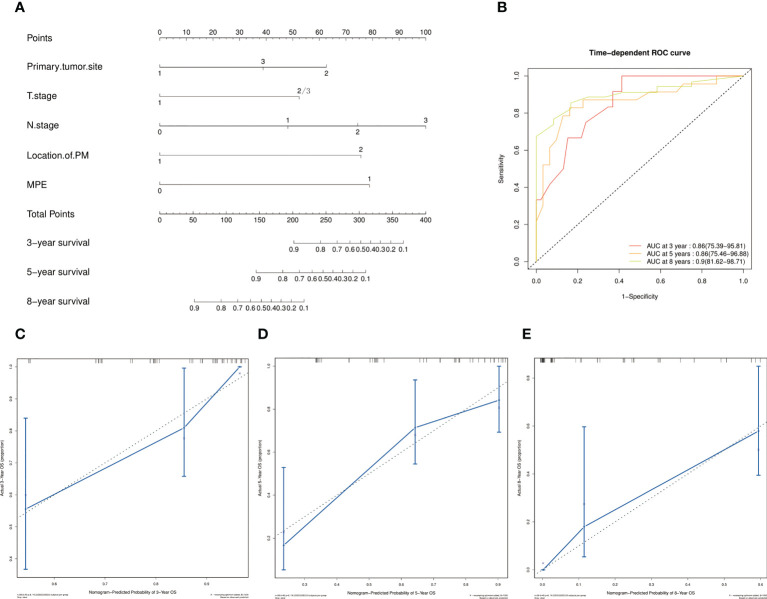
Prognostic nomograms of 3-, 5-, and 8-year M-OS in patients with only PM at first MBC diagnosis. Points are defined based on the prognostic contribution of the factors. Points summing the contribution of Primary tumor site, T Stage, N Stage, Location of PM, and MPE are translated to the survival probability at 3, 5 and 8 years **(A)**; ROC curve with AUC for 3-, 5-, and 8-year M-OS rate in patients with solitary PM at first MBC diagnosis **(B)**; Calibration curves of the nomogram for 3-, 5-, and 8-year M-OS prediction **(C–E)**.

The screened five factors were used to develop a nomogram for patients with only PM at first MBC diagnosis (**Figure 5A**), and all the predictors were integrated to predict the 3-, 5-, and 8-year M-OS of patients. The nomogram exhibited favorable accuracy in predicting the M-OS with a C-index of 0.776 (95% CI = 0.740–0.812). The above outcomes corresponded with the ROC curves and AUC values (**Figure 5B**). The AUC values of 3-, 5-, and 8-year M-OS were 0.86, 0.86, and 0.90, respectively, which were > 0.70, indicating that the constructed nomogram has good predictive efficiency for M-OS. The calibration curves revealed that the predictive outcomes were in good accordance with the actual 3-, 5-, and 8-year M-OS (**Figures 5C–E**).

## 4 Discussion

Tumor metastasis contributes to high cancer mortality (30), BC has variable aggressiveness and a high propensity to develop distant metastases (31). Extensive studies have proven that BC exhibits metastatic heterogeneity with distinct metastatic precedence to various organs, leading to differences in responses to therapy and prognoses (1). Recent studies have revealed that BC subtypes differ not only in primary tumor characteristics but also in their metastatic behavior (32). In our study, although there was no significant difference in the molecular subtype of primary cancer among the three groups, PM, LM, and PM combined with LM mainly originated from luminal B (39.7%), triple negative (30.9%), and luminal B (34.9%) types, respectively.

The first site of distant metastasis is associated with the prognosis of BC patients (6). Although pleura is a common metastatic site of BC (7), PM has rarely been reported as the first metastatic site in patients with BC. The proportion of such patients may be underestimated because of the time lag in follow-up examinations or the lack of accurate and effective means of examination. In our research, 29.3% of patients did not visit the hospital until presenting with symptoms related to PM. The prognosis of BC patients with single-site metastasis was significantly better than that of patients with multiple metastatic sites (33). In addition, the presence of visceral metastases has a significant negative prognostic impact on patients (28). Schröder J et al. revealed that patients with bone-only metastasis showed better survival than visceral with or without bone metastases (34). Our results indicated that the prognosis of patients with PM not complicated by other distant sites is indeed better than that of patients combined with LM or whose lung serve as the single distant metastatic site. Despite no significant advantage was observed in M-OS for patients with only LM compared with patients with combined PM, the survival rates at 3-, 5- years were all superior. Despite improvements in treatment, MBC has a poor prognosis and an overall 5-year survival rate of only 27% for patients in the United States (35). However, LM has a relatively good prognosis in visceral metastasis as the first distant metastasis of BC (6, 13). Redig AJ et al. tested the relationship between site of metastasis and outcome, and the best prognosis was observed among patients with lung as first anatomic site of distant metastasis, followed by those with first metastatic involvement of bone, liver and central nervous system (6). Combined the existing data, it might be inferred that PM has a better prognosis than visceral metastasis. However, further validation with clinical data is required and the underlying mechanism should be elucidated.

PM most commonly originates from metastatic lung carcinomas and breast carcinomas (36); the mechanisms include hematogenous spread, direct invasion from a neighboring cancer, and retrograde lymphatic spread from the mediastinum (37). Breast carcinoma is the most common metastatic malignancy identified in pleural effusion specimens from women (38). PM is often accompanied by MPE (39), but not all tumors metastasizing to the pleura cause MPE (40). In our results, the incidence of MPE in patients with PM (81.0% in group A and 76.7% in group C) was high. On the one hand, MPE is consider an unfavorable complication that restricts life quality (41) and related to poor prognosis (42). Consistently, our study reported that in BC, MPE was an independent risk factor for patients with PM. On the other hand, Poe RH et al. reported that the median survival of BC patients in whom MPE was the initial and only recurrent site was 48 months, compared with 12 months for patients associated with other metastatic diseases (43). The MPE is more commonly unilateral and ipsilateral to the primary BC (18), Poe RH et al. suggested that this indicated that MPE was a regional rather than systemic disease, probably accounting for the better outlook in patients with effusion alone (43). Similarly, our data showed that the majority of initial PM was located ipsilateral and had a better prognosis. PM located contralateral/bilateral to the primary BC is a factor that worsens the prognosis. Differently, the patients with MPE without other distant metastases at the initial diagnosis of MBC exhibited a better prognosis compared with LM patients with or without PM, but without significant difference (**Supplemental Figure 2**). Whereas the M-OS of PM patients without MPE was substantially longer (median M-OS:89.3 months in group A; 64.0 months in group C). Thomas et al. (44) speculated that in BC, the laterality of PM is because of lymphatic dissemination. Similarly, Agalioti T et al. (39) stated that BC may invade the pleura because of local proximity rather than through the bloodstream. This may be one of the reasons for its better prognosis than other distant metastases. Moreover, pleura is of itself innocuous and once thought to be biologically inert (45). Oncogene signals and/or transcription factor activation in tumor cells determine paracrine gene expression. The balance between vasoactive mediators and possible protective molecules in the pleural space dictates the occurrence of vasoactive signaling with subsequent MPE development. In turn, this signal cocktail exert a multitude of effects on tumor cells (46). To some extent, tumor colonization of the pleura but not causing MPE may be a manifestation of its poor malignant biological behavior. This is also reflected by other clinical features of these patients. Patients with ipsilateral PM without MPE as their only evidence of distant metastasis may could to be staged as limited disease. However, our data are limited and potentially biased. More clinical data and the specific mechanism investigation are needed in the future for further elucidation.

Our study classified patients into three groups according to primary cancer location: outer quadrant, inner quadrant, and areolar area/central axis. Pokieser W et al. (47) reported that invasive ductal carcinomas located in the inner quadrants were significantly associated with increased pleural effusion as the first site of metastasis, which may be associated with a higher rate of internal mammary lymph node metastasis. Similarly, our study reported that 46.6% of patients had primary cancers located in the inner quadrant. Furthermore, our results indicated that primary cancer location in the inner quadrant is a poor prognostic factor for patients. Some studies demonstrated that BC situated in inner quadrants have a worse prognosis (48–51), which may cause by the anatomical accessibility of the tumor to the internal mammary lymph node (49, 52). Additionally, growing evidence suggests differences in metastatic spread among BC biologic subtypes (6). Smid M et al. suggested that the majority of pleural relapse occurred in both luminal subtypes (53), which is consistent with our findings. Prognosis of metastatic breast is confirmed to be affected by a combination of factors such as molecular features (54). The prognostic role exerted by pathological factors varies in different disease contexts. Although we observed significant differences in M-OS among the four molecular subtypes of BC (**Supplemental Figures 3A, B** **)**, multivariate Cox results revealed that it was not an independent prognostic factor for patients with only PM. Similarly, Yang Y et al. suggested that the prognosis of patients with cancer with MPE was independent of histology (41). This may be caused by the particularities of the studied patients or by data bias. In addition, BC is highly heterogeneous, and patients with the same molecular subtype also have distinct molecular features, responses to treatment, and prognosis (55, 56). Global burden of molecular mutations into primary tumor and metastatic samples seemed to be independent of the molecular subtype of primary tumor and metastatic sites in the study of Callens C et al. (54). By contrast, one study by Schrijver et al. showed different molecular mutational signatures for different metastatic sites (57). This may be one of the reasons why molecular subtypes did not appear as a predictor of survival in PM patients without other distant metastases, and the mechanisms remain to be further investigated and elucidated. Furthermore, the lymph node status and tumor size were independent predictors of death due to BC (58). Several studies have reported that the higher the T/N stage, the worse the prognosis of patients with BC (59), which was consistent with our results.

Evidence-based guidelines suggest the use of conservative treatments for patients with limited life expectancy, whereas they suggest offering more aggressive treatment modalities for patients with better prognoses. In this study, we focused on analyzing the survival of patients with PM without other distant metastasis at the time of first MBC diagnosis and identifying the prognostic factors. Identifying these characteristics and understanding their prognostic value in diseases could enable customized treatments for this patient group. The nomogram model constructed in this study included all the independent risk factors that we screened, and it provided a visual and user-friendly tool for risk evaluation and prognostic prediction of patients with BC with only PM, facilitating tailored management strategy for these patients.

However, inevitable the study has some limitations. (1) Our study was a single-center retrospective analysis with a limited number of cases, which may have caused some restrictions and biases in the results. (2) Although the nomogram achieved ideal prediction efficacy; it lacked external validation to further enforce the reliability.

## 5 Conclusion

BC with PM without additional distant metastasis at the time of first MBC diagnosis exhibited a better prognosis than those with combined LM or LM alone. For patients with PM, the prognosis of patients with MPE was worse. Primary cancer site, T stage, N stage, location of PM, and MPE were identified as independent prognostic factors for predicting M-OS in patients with PM as the only distant metastatic site. The nomogram provided a quantitative method for predicting individual survival in this subset of patients.

## Data availability statement

The raw data supporting the conclusions of this article will be made available by the authors, without undue reservation.

## Author contributions

SL, ZY, and CL contributed to the conception and design of the study. SL collected data, performed the statistical analysis, and wrote manuscript. CL, WS, XL, and LS wrote sections of the manuscript. ZY reviewed and revised the manuscript, and acts as guarantor. All authors listed have read the final manuscript and agree to its publication.
