# Supplementary material for: Survival analysis and prognosis of patients with breast cancer with pleural metastasis
Source: Front Oncol. 2023 May 1;13:1104246. doi: 10.3389/fonc.2023.1104246 (PMC10183576; doi:10.3389/fonc.2023.1104246)
Supplement: Supplementary file 1 [file DataSheet_1.docx]

## Supplementary Figures

**Figure 1.**


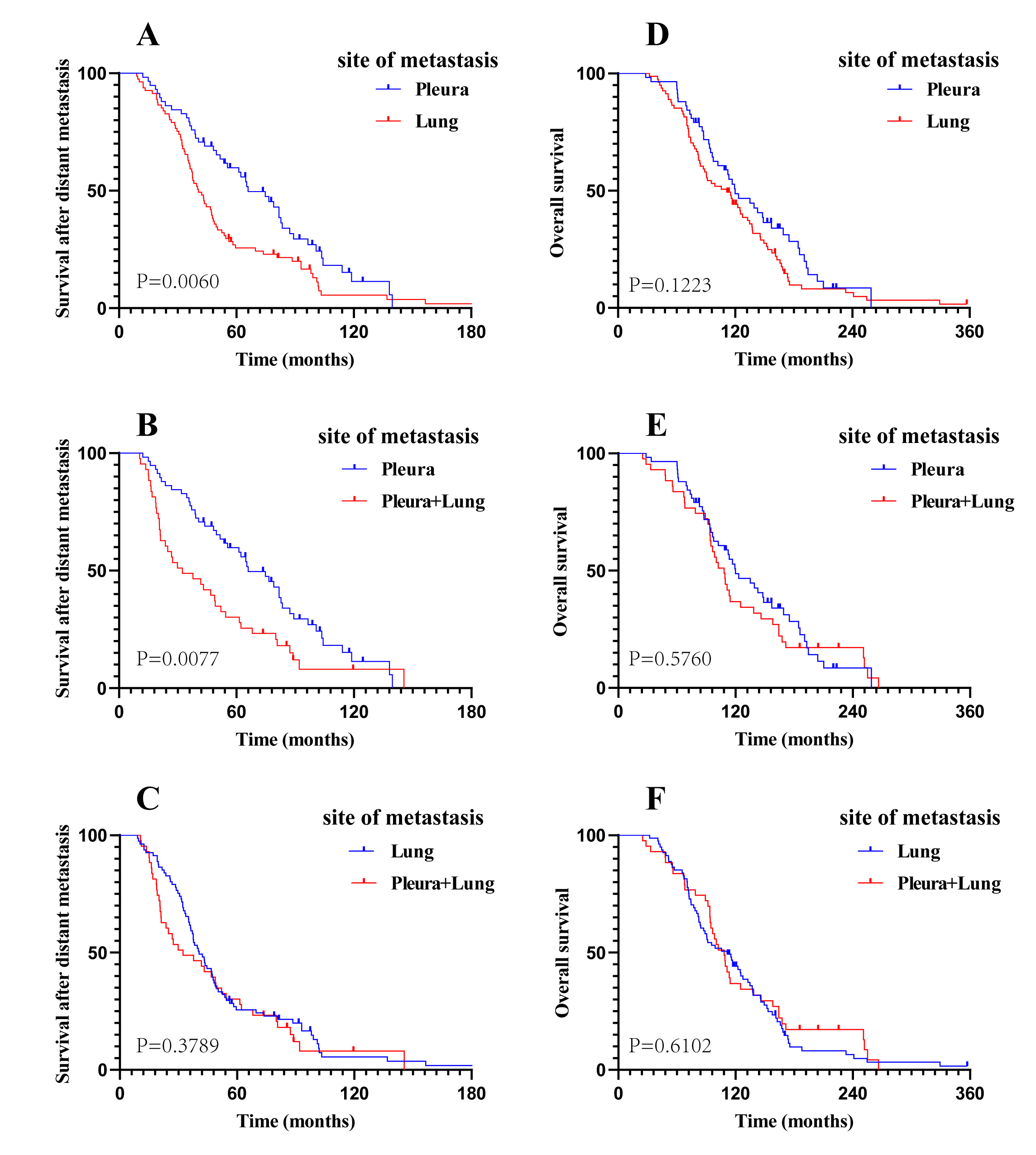


**Figure 1.** The Kaplan–Meier curve analysis of study cohorts. M-OS (A-C) and OS (D-F) curves according to different metastatic sites at the time of diagnosed of metastatic breast cancer. M-OS: survival after distant metastasis; OS: overall survival.

**Figure 2.**


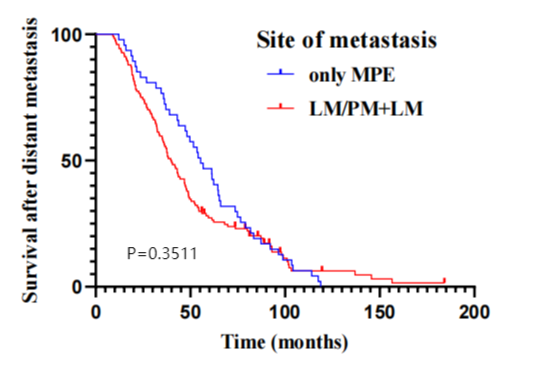


**Figure 2.** Survival analysis of M-OS in relation to MPE. M-OS: survival after diagnosis of distant metastasis; only MPE: MPE without other distant metastasis; LM/PM+LM: LM with or without PM.

**Figure 3.**


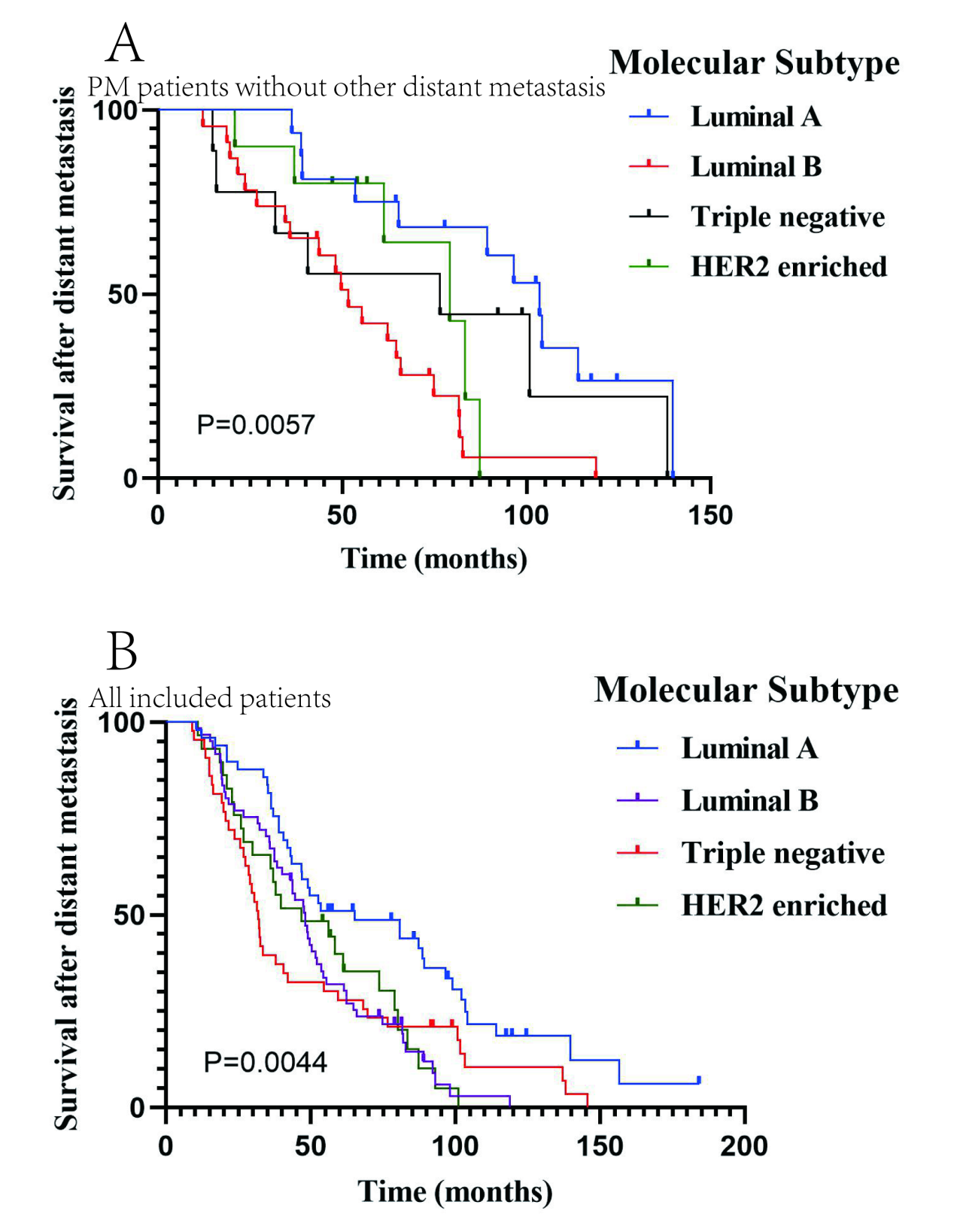


**Figure 3.** Survival analysis of M-OS in PM patients without other distant metastasis (A) and all included patients (B) according to molecular subtype.
